# Interferon-based HBV function cure therapy reduces hepatocellular carcinoma risk in low-HBsAg chronic hepatitis B patients: a propensity-matched cohort study and dynamic prediction model

**DOI:** 10.3389/fimmu.2026.1955742

**Published:** 2026-10-19

**Authors:** Yurong Gu, Peipei Wang, Qinyao Xu, Ting Liu, Huaping Xie, Baoer Wu, Yusheng Jie, Xiangyong Li

**Affiliations:** 1 Department of Infectious Diseases, the Third Affiliated Hospital of Sun Yat-sen University, Guangzhou, China; 2 Guangdong Provincial Key Laboratory of Liver Disease Research, The Third Affiliated Hospital of Sun Yat-sen University, Guangzhou, China; 3 The Second School of Clinical Medicine, Southern Medical University, Guangzhou, Guangdong, China

**Keywords:** functional cure, hepatitis B, hepatocellular carcinoma, interferon, prediction model, propensity score matching

## Abstract

**Background:**

Functional cure through HBsAg loss is a key treatment goal in chronic hepatitis B (CHB), yet its impact on hepatocellular carcinoma (HCC) risk remains incompletely characterized, especially when achieved via interferon (IFN)-based therapy. We aimed to evaluate the HCC risk reduction associated with IFN-based therapy in low-HBsAg CHB patients and develop a validated prediction model for this population.

**Methods:**

We conducted a retrospective cohort study of 3,367 CHB patients with HBsAg <1,500 IU/mL, including 1,780 receiving IFN-based therapy and 1,587 on nucleos(t)ide analogue (NAs) monotherapy. Propensity score matching (PSM) was used to balance baseline characteristics. Cox regression and time-dependent ROC analyses were employed to develop a dynamic nomogram for HCC prediction.

**Results:**

IFN-based therapy was associated with a significantly lower HCC incidence compared to NAs monotherapy (1.2% vs. 4.9%, p<0.001), with a hazard ratio of 3.03 (95% CI: 1.82–5.06) after PSM. The protective effect was most pronounced in cirrhotic patients (absolute risk reduction 8.8%, NNT = 11). A novel nomogram incorporating treatment modality, AFP, cirrhosis, age, and other variables demonstrated excellent predictive accuracy (5-year AUC: 0.888 in derivation, 0.956 in validation cohorts), outperforming existing HCC prediction models.

**Conclusions:**

IFN-based therapy significantly reduces HCC risk in low-HBsAg CHB patients, particularly those with cirrhosis. We developed and validated a dynamic prediction model that may facilitate personalized surveillance and treatment decisions in functionally cured patients.

## Introduction

Hepatitis B virus (HBV) infection poses a major global health burden, with an estimated 254 million people living with chronic hepatitis B (CHB) in 2022 and approximately 1.1 million annual HBV-related deaths, largely attributable to hepatocellular carcinoma (HCC) (1–3). HCC, the most severe long-term consequence of CHB, is characterized by high mortality, aggressive biology, frequent late diagnosis, and limited curative options beyond early detection (4).

HBV infection increases HCC risk 100-200-fold compared to uninfected individuals (5), though risk varies substantially with disease activity, viral load, and fibrosis stage (6). This heterogeneity underscores the critical need for precise risk stratification tools (7). Established models like REACH-B, CU-HCC, GAG-HCC, and mPAGE-B, primarily derived from untreated or nucleos(t)ide analogue (NA)-treated cohorts, have significantly improved risk prediction and guided surveillance in general CHB populations (8–12).

The current paradigm of CHB management prioritizes functional cure—defined as sustained HBsAg loss with or without HBsAb seroconversion—which is associated with improved clinical outcomes, including a reduced risk of HCC (13). Interferon (IFN)-based therapies play a pivotal role in achieving this therapeutic endpoint (14). The immunomodulatory and virological changes induced by IFN-based treatment, particularly when leading to functional cure, may fundamentally alter the long-term risk profile of CHB patients. However, it is important to note that achieving functional cure does not entirely eliminate HCC risk, especially in patients with pre-existing cirrhosis or advanced fibrosis (15). Moreover, current IFN-based regimens—whether used as monotherapy or in combination with nucleos(t)ide analogues—yield functional cure rates below 40%, even in selected advantage populations. This indicates that a substantial proportion of patients receiving IFN-based therapy do not achieve HBsAg clearance, and how IFN treatment influences HCC risk in this non-cured subgroup remains poorly characterized. Critically, existing HCC prediction models were primarily derived from untreated or nucleos(t)ide analogue (NA)-treated cohorts, and their performance has not been adequately validated in the distinct and growing population of patients treated with IFN-based regimens. Although several studies have reported HCC incidence after HBsAg loss (15, 16), they often combine patients who achieved functional cure through different pathways (spontaneous, NA-induced, or IFN-induced) and lack dedicated risk stratification tools specifically tailored for the IFN-treated subgroup—a limitation frequently compounded by small sample sizes and insufficient follow-up duration (16, 17).

Consequently, a validated HCC risk prediction model tailored for CHB patients achieving functional cure through IFN therapy is urgently needed to optimize post-cure surveillance and long-term outcomes. To address this gap, we developed and validated a novel HCC prediction model using a large cohort of CHB patients who attained functional cure following Peg-IFN-based therapy, with comprehensive clinical, virological, and serological data collected during and up to 5 years post-therapy.

## Methods

### Patients

This retrospective cohort study was conducted at the Third Affiliated Hospital of Sun Yat-sen University (Guangzhou, China). Between January 2018 and December 2024, we enrolled two treatment-defined cohorts of CHB patients: 1) nucleos(t)ide analogue (NA) monotherapy cohort, and 2) a functional cure cohort comprising patients who achieved sustained HBsAg loss following NA plus pegylated interferon (Peg-IFN) combination therapy.

All participants met identical inclusion criteria defined per functional cure optimization guidelines (18), requiring chronic HBsAg positivity (≥6 months) with baseline HBsAg <1,500 IU/mL, HBeAg-negative status (including patients who seroconverted during prior NA therapy), undetectable HBV DNA at enrollment, fulfillment of 2022 Chinese guideline antiviral criteria (18), and age 18–65 years. Exclusion criteria applied uniformly across cohorts and comprised coinfection (HAV, HCV, HDV, HIV), decompensated cirrhosis, significant alcohol consumption (>30g/day for men; >20g/day for women), active illicit drug use, autoimmune or metabolic liver diseases, extrahepatic malignancies, severe extrahepatic organ dysfunction, and pregnancy or lactation.

### Treatment and follow-up

Patients in the NA monotherapy cohort maintained continuous oral NA therapy (entecavir (ETV) or tenofovir formulations (TDF/TAF)) throughout the follow-up period. Patients in NA plus pegylated interferon cohort received combination therapy comprising NAs plus subcutaneous pegylated interferon-α (Peg-IFNα-2b, Pegbing^®^, Xiamen Amoytop Biotech Co., Ltd.) at a dose of 180 μg/week for 48–96 weeks, followed by NA maintenance after achieving sustained HBsAg loss. Follow-up commenced at cohort entry for NA monotherapy patients and at confirmed sustained HBsAg loss for the functional cure cohort, continuing until HCC diagnosis, last documented clinical encounter (for censored cases), or study conclusion (December 31, 2024), whichever occurred first.

Comprehensive data collection encompassed baseline and serial measurements of demographic characteristics (age, sex, BMI), clinical history (family history of HBV/HCC, alcohol consumption patterns), laboratory parameters (quantitative HBsAg, anti-HBs, HBeAg, anti-HBe, AFP, ALT, AST, albumin, total protein, total/direct bilirubin, complete blood counts), and hepatic status indicators (liver stiffness, cirrhosis confirmation through histopathology or imaging/clinical criteria). Standardized abdominal ultrasound examinations were performed at 6-month intervals, with contrast-enhanced CT or MRI conducted when clinically warranted.

Patients underwent scheduled follow-up evaluations every 3–6 months, during which we assessed therapeutic responses including ALT normalization (<40 U/L), HBsAg seroclearance (confirmed by>=2 consecutive negative tests), HBsAg seroconversion (anti-HBs >=10 mIU/mL), and HBeAg seroconversion. The primary study endpoint was HCC development.

### Definitions

The diagnosis of liver cirrhosis was established histopathologically when biopsy specimens were available; otherwise, it was confirmed through composite imaging and clinical criteria comprising ultrasonographic features of coarse echotexture with nodularity and/or morphological alterations coupled with clinical evidence of portal hypertension (including ascites, splenomegaly, or documented esophageal varices) (19, 20). HCC was diagnosed according to histopathological examination or characteristic radiological features on multiphasic contrast-enhanced CT or MRI, consistent with National Comprehensive Cancer Network (NCCN) guidelines (LI-RADS 5 category or equivalent) (8). In cases where both pathological and radiological assessments were available, histological confirmation took precedence.

### Ethical approval

The study protocol received approval from the Institutional Review Board of the Third Affiliated Hospital of Sun Yat-sen University (Approval No. II2023-058-02). The requirement for informed consent was waived due to the retrospective design and use of anonymized clinical data in accordance with the Declaration of Helsinki. All patient identifiers were removed prior to data analysis.

### Statistical analyses

Normality of all continuous variables were checked by Kolmogorov–Smirnov test and reported as mean ± standard deviation or median (PR25, PR75). When comparing continuous variables between the NAs and IFN treatment groups, Mann-Whitney U test was used. Categorical variables were presented as number and percentage and were compared using Chi-square test or Fisher’s exact text (if expected value <=5 was found). Propensity score matching (PSM) procedure was used to select paired cases from two groups while multiple baseline variables were controlled. This new subset data after PSM was applied to most following analyses. The matching method is “nearest”, sample size ratio between groups was set at 1, and the caliper value was 0.2. The R package ‘MatchIt’ were used to perform PSM. The standardized mean difference (SMD) before and after PSM was provided to confirm the change of bias after PSM.

To further investigate the associations between independent variables and the occurrence of HCC, the univariate and multivariate logistic regression models were used. The independent variables which were significant in univariate results would be entered into the multivariate logistic regression model with forward (Wald statistic) selection procedure; and the independent variables which were reported significant in multivariate model would be recognized as associated factors to the risk of occurring HCC.

All analyses were done using statistical software R (version 4.5.0). The statistical significance level for all the tests was set at a P-value < 0.05, two-tailed.

## Results

### Patient characteristics

A total of 3,880 CHB patients with low HBsAg levels were initially screened. After excluding 513 patients who met predefined exclusion criteria, 3,367 patients were enrolled in the final cohort. Of these, 1,587 received NAs monotherapy (NAs group), while 1,780 received combination therapy with NAs and interferon (IFN group). All patients were monitored for clinical outcomes over a 6-year follow-up period. The participant flow is illustrated in **Figure 1**.

**Figure 1:**
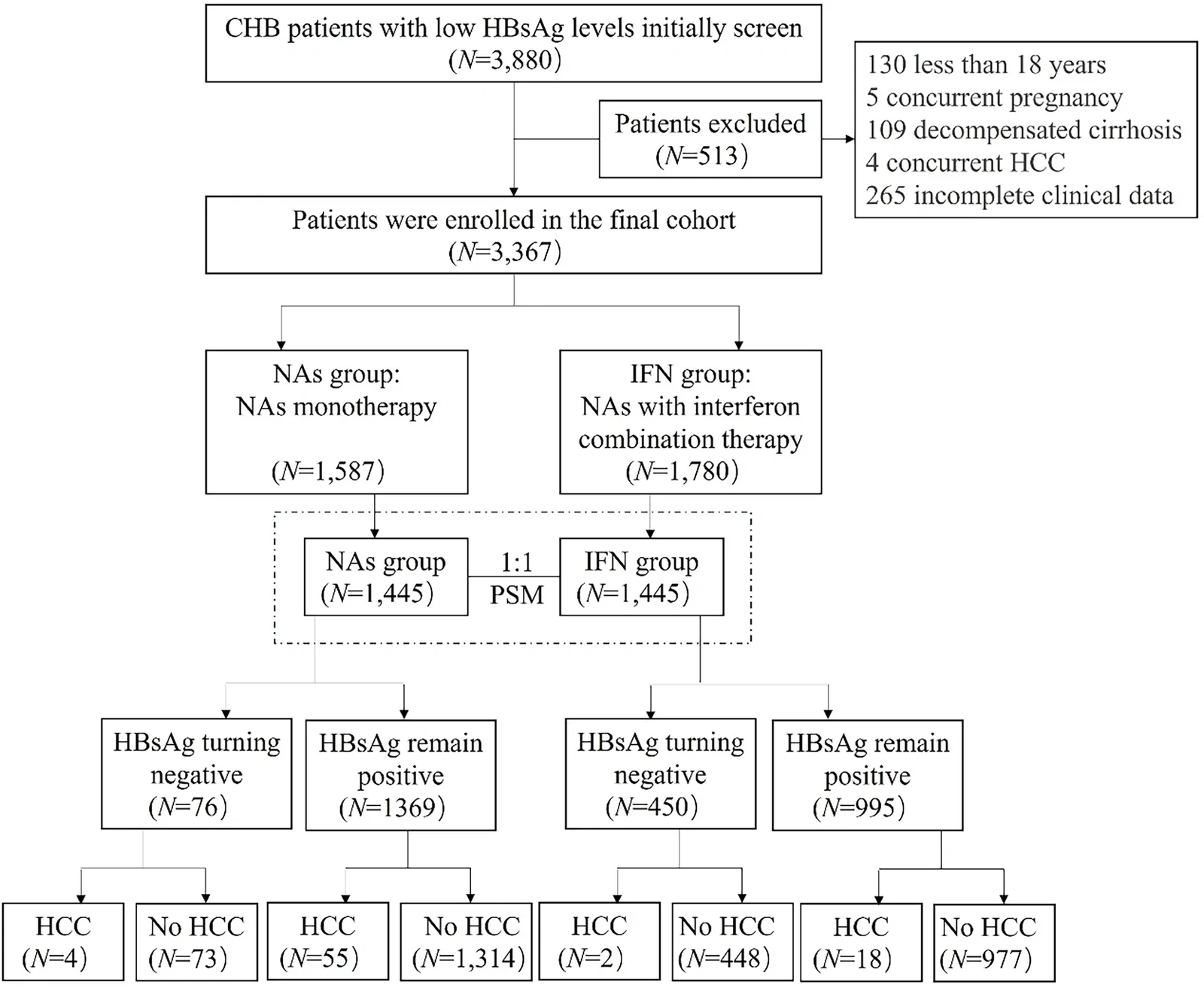
Flow chat of this study.

Baseline characteristics revealed imbalances between two groups (**Supplementary Table 1**). The IFN group was younger, with fewer females, lower rates of cirrhosis and family history of HCC, and distinct laboratory profiles including higher AFP, PLT, HGB and ALB but lower AST, GGT, TBIL, DBIL, GPR, FIB-4, and APRI levels (all p<0.05). Larger left liver thickness and longer spleen length were also observed in NAs group.

After treatment, significantly more rates of IFN group patients with HBsAg turning negative, but less rates of IFN group patients with positive HBsAb, ALT returning normal, ALT level rising, and with occurrence of HCC.

To control for the various baseline differences, we performed a 1:1 PSM adjustment using key HCC risk factors: age, sex, cirrhosis status, family history of HCC, baseline HBsAg, ALT, and AFP levels. This yielded 1,445 matched pairs (n=2,890 total). Standardized mean differences (SMD) for matched covariates were reduced to <0.05 post-PSM (**Supplementary Figure 1**), indicating adequate balance for these variables. **Table 1** presents the new intergroup comparison results after above PSM. As indicated in **Figure 1**, the SMDs of adjusted variables all reduced to acceptable level (<0.05) after PSM. However, due to the continuous variables not conforming to the assumption of normality, **Table 1** reports the results non-parametric statistics and tests, which differ from the parametrical viewpoint of calculating SMD. Therefore, although PSM was successful, there are still differences between the two groups in AFP and ALT according to the results of the non-parametric tests.

**Table 1:** The descriptive results between NAs and IFN treatment groups after PSM.

Variable	NAs	IFN	All	P
n	1445	1445	2890	
Age, year	41 (35, 49)	43 (37, 49)	43 (36, 49)	0.051
Sex, female	301 (20.8%)	275 (19.0%)	576 (19.9%)	0.244
BMI group				0.506
0	2 (0.1%)	0 (0.0%)	2 (0.1%)	
1	1108 (76.7%)	1097 (75.9%)	2205 (76.3%)	
2	296 (20.5%)	306 (21.2%)	602 (20.8%)	
3	39 (2.7%)	42 (2.9%)	81 (2.8%)	
Diabetes mellitus	13 (0.9%)	8 (0.6%)	21 (0.7%)	0.381
Fatty liver	29 (2.0%)	405 (28.0%)	434 (15.0%)	<0.001
Family history of HepaB	845 (58.5%)	563 (39.0%)	1408 (48.7%)	<0.001
Family history of HCC	97 (6.7%)	82 (5.7%)	179 (6.2%)	0.280
Drink	273 (18.9%)	22 (1.5%)	295 (10.2%)	<0.001
Cirrhosis	215 (14.9%)	193 (13.4%)	408 (14.1%)	0.262
AFP	2 (1, 3)	3 (2, 4)	2 (2, 3)	<0.001
ALT	26 (19, 37)	24 (18, 34)	25 (19, 35)	0.001
WBC	6 (5.03, 6.97)	6 (5, 7.09)	6 (5.02, 7.05)	0.879
PLT	196 (160, 243)	210 (174, 246)	205 (167, 245)	<0.001
NEUT	3.25 (2.60, 4.05)	3.28 (2.55, 4.10)	3.27 (2.58, 4.08)	0.933
HGB	152 (139, 160.50)	153 (143, 162)	153 (141, 161)	<0.001
AST	25 (21, 31)	24 (20, 28)	25 (21, 30)	<0.001
ALB	46.40 (44.50, 48.30)	47.90 (46.20, 49.70)	47.20 (45.30, 49)	<0.001
GLB	28.02 (25.60, 30.40)	28.30 (26.10, 30.70)	28.20 (25.80, 30.50)	0.015
GGT	25 (17, 38)	23 (17, 35)	24 (17, 37)	0.022
TBIL	13.30 (9.90, 18.25)	11.20 (8.50, 14.60)	12.20 (9.10, 16.40)	<0.001
DBIL	3.80 (2.70, 5.40)	2.70 (2, 3.80)	3.20 (2.30, 4.60)	<0.001
HbsAg (QN)	404 (126.35, 801.30)	379 (122, 744)	392.70 (122.30, 780.73)	0.383
GPR	0.21 (0.13, 0.36)	0.20 (0.13, 0.30)	0.20 (0.13, 0.33)	0.001
FIB4 group				<0.001
1	1003 (69.4%)	1043 (72.2%)	2046 (70.8%)	
2	329 (22.8%)	368 (25.5%)	697 (24.1%)	
3	113 (7.8%)	34 (2.4%)	147 (5.1%)	
APRI group				<0.001
1	1091 (75.5%)	1210 (83.7%)	2301 (79.6%)	
2	301 (20.8%)	224 (15.5%)	525 (18.2%)	
3	53 (3.7%)	11 (0.8%)	64 (2.2%)	
Virus load > 2 log	1120 (77.5%)	1121 (77.6%)	2241 (77.5%)	1.000
Left liver thickness	110 (104, 118)	108 (103, 114)	110 (104, 116)	<0.001
Left liver anteroposterior diameter	55 (46, 63)	54 (45, 62)	55 (46, 62)	0.178
Left liver lobe superior inferior dimension	65 (53, 76)	65 (53, 76)	65 (53, 76)	0.776
Portal vein diameter	11 (10.85, 12)	11 (10.80, 12)	11 (10.80, 12)	<0.001
Spleen length	100 (90.50, 111)	98 (89, 109)	99 (90, 110)	<0.001
Splenic hilum vein diameter	6 (5, 6)	6 (5, 6)	6 (5, 6)	0.897
HBsAg turning negative	76 (5.3%)	450 (31.1%)	526 (18.2%)	<0.001
HBsAb, positive	1428 (98.8%)	998 (69.1%)	2426 (83.9%)	<0.001
HBeAb, positive	1273 (88.1%)	1234 (85.4%)	2507 (86.7%)	0.037
ALT returning normal	1337 (92.5%)	1274 (88.2%)	2611 (90.3%)	<0.001
ALT level rising	406 (28.1%)	339 (23.5%)	745 (25.8%)	0.005
AFP at normal level	1389 (96.1%)	1361 (94.2%)	2750 (95.2%)	0.019
HCC	59 (4.1%)	20 (1.4%)	79 (2.7%)	<0.001

As shown in **Table 1**, the IFN group exhibited a higher incidence of fatty liver, fewer family histories of HBV, and fewer patients with alcohol consumption habits. In terms of laboratory results, the IFN group had significantly higher levels of AFP, PLT, HGB, ALB, GLB, and significantly lower levels of ALT, AST, GGT, TBIL, DBIL, GPR, FIB4, APRI. The NAs group still presented with greater left liver thickness, longer spleen length, and larger portal vein diameter.

After treatment, the IFN group had a higher number of patients with HBsAg turning negative, fewer patients maintaining positive HBsAb, positive HBeAb, ALT returning to normal, rising ALT levels, AFP at normal levels, and the occurrence of HCC.

### Cumulative incidence of HCC

#### HCC incidence and cumulative risk in primary cohorts

During the 6-year follow-up, 98 incident HCC cases were documented. The interferon-based combination therapy group (NAs+IFN, n=1,780) demonstrated significantly lower HCC incidence (21 cases, 1.2%) compared to the nucleos(t)ide analogue monotherapy group (NAs, n=1,583; 77 cases, 4.9%), representing a 4.27-fold risk difference (95% CI 2.62-6.95, p<0.001).

Kaplan-Meier analysis of the pre-PSM cohort revealed substantially superior HCC-free survival in IFN-treated patients (log-rank p<0.001; **Supplementary Figure 2**). The cumulative incidence curves demonstrated progressive divergence between treatment arms beginning at approximately 96 weeks, with the protective effect of IFN-based therapy becoming increasingly prominent throughout the 6-year observation period. By week 288, the absolute difference in cumulative event rates exceeded 3.5%, corresponding to a number needed to treat of 29 to prevent one HCC event within six years.

#### HCC incidence and cumulative risk in PSM cohorts

In after-PSM cohort, HCC incidence remained significantly elevated in the NAs group (59 cases, 4.1%) versus the IFN group (20 cases, 1.4%), with a persistent 3.03-fold risk increase (95% CI 1.82-5.06, p<0.001). Kaplan-Meier analysis of the matched cohort confirmed significantly superior HCC-free survival in the IFN-based therapy group (log-rank p<0.001; **Figure 2**). The cumulative incidence curves showed sustained separation throughout the follow-up period, with the treatment benefit emerging after approximately 48 weeks and maintaining consistent divergence through 288 weeks. The absolute risk reduction with IFN-based therapy was 2.7% in the matched cohort, corresponding to a number needed to treat of 37 to prevent one HCC event over 6 years.

**Figure 2:**
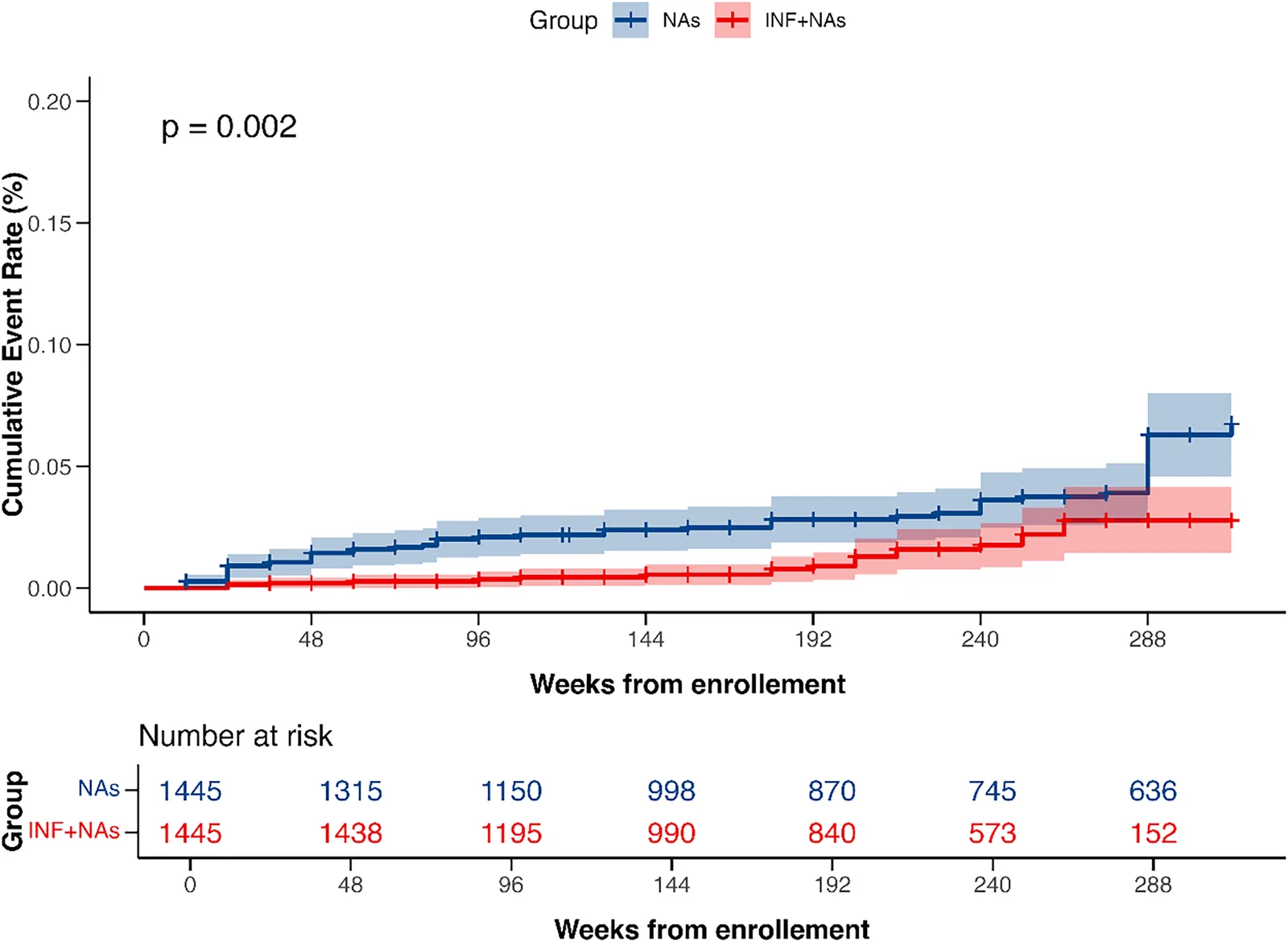
Kaplan-Meier analysis of HCC-free survival in the PSM cohort. Cumulative incidence of hepatocellular carcinoma in patients receiving nucleos(t)ide analog (NAs) monotherapy (blue line) versus interferon-based combination therapy (NAs + IFN, red line) after propensity score matching (n=2,890, 1:1 matched). Log-rank test demonstrated significantly superior HCC-free survival in the NAs + IFN group (p < 0.001). Numbers at risk at each time point are shown below the figure. Follow-up duration is shown in weeks (x-axis).

#### HCC incidence stratified by cirrhosis status

##### HCC incidence in non-cirrhotic patients

In primary cohorts of non-cirrhotic patients (n=2,896), IFN-based combination therapy demonstrated significant protection against HCC development. Kaplan-Meier analysis revealed superior HCC-free survival in the IFN group compared to NAs monotherapy (log-rank p=0.019; **Supplementary Figure 3**). The cumulative incidence curves showed progressive separation between treatment arms, with the IFN group maintaining a lower event rate throughout the 6-year follow-up period.

After PSM of non-cirrhotic patients (n=2,482, 1,241 per group), the protective effect of IFN-based therapy, while still evident in the cumulative incidence curves, did not reach statistical significance in the time-to-event analysis (log-rank p=0.110; **Figure 3**). The attenuation of statistical significance after matching suggests that the observed benefit in the pre-matched analysis may have been influenced by baseline imbalances rather than representing a pure treatment effect in non-cirrhotic patients.

**Figure 3:**
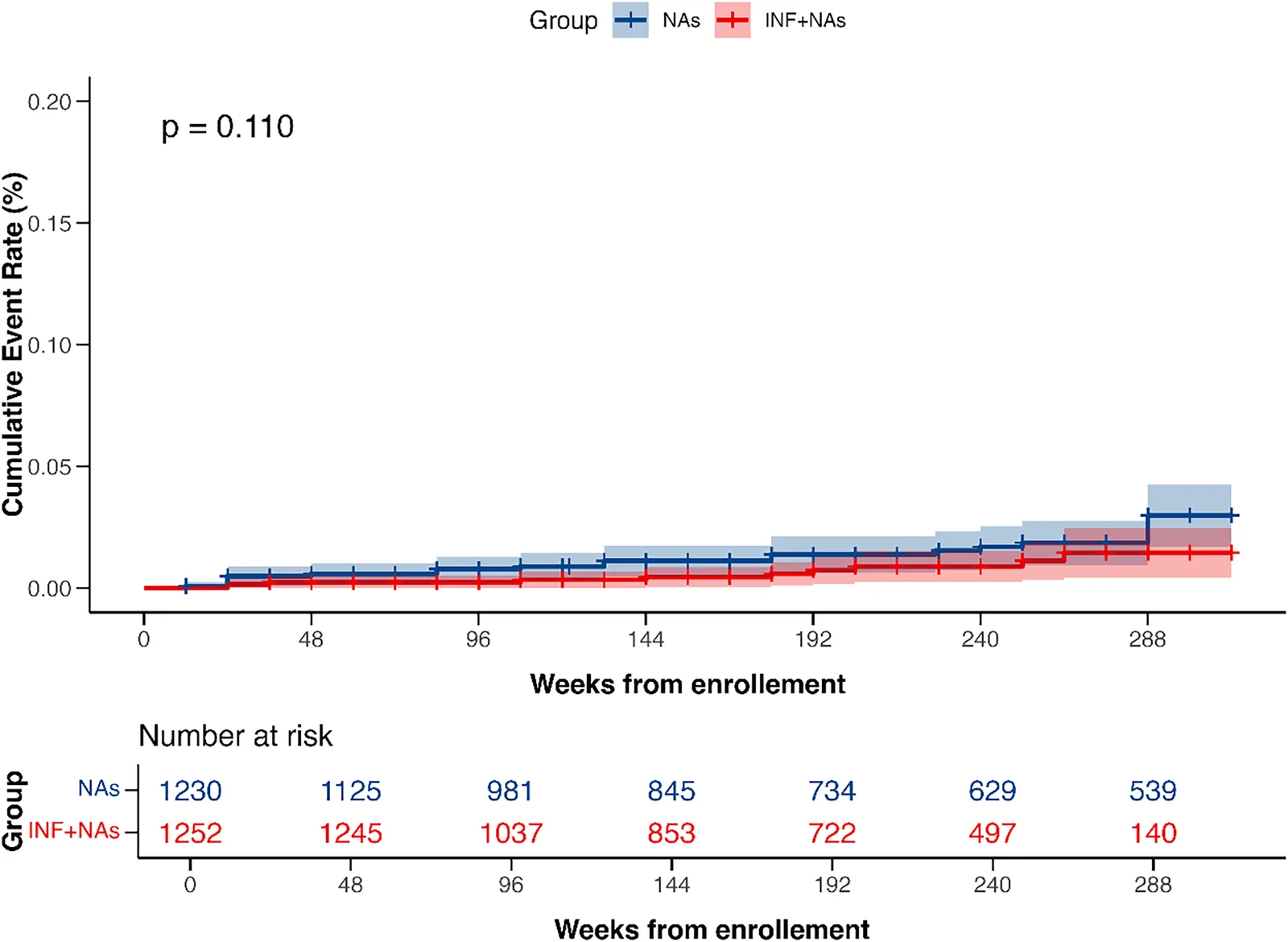
Kaplan-Meier analysis of hepatocellular carcinoma-free survival in non-cirrhotic patients before PSM. Cumulative incidence of hepatocellular carcinoma in non-cirrhotic patients receiving nucleos(t)ide analog (NAs) monotherapy (red line) versus interferon-based combination therapy (NAs + IFN, blue line) in the pre-matched cohort (n=2,896). Log-rank test demonstrated significantly superior HCC-free survival in the NAs + IFN group (p = 0.019). Numbers at risk at each time point are shown below the figure.

The absolute HCC incidence rates in the matched non-cirrhotic cohort were numerically lower in the IFN group (3.2% vs 4.1% in NAs group), representing a 0.9% absolute risk reduction, though this difference did not achieve statistical significance (p=0.085). The number needed to treat to prevent one HCC event in non-cirrhotic patients was 111 over 6 years.

##### HCC incidence in cirrhotic patients: enhanced risk and treatment benefits

Cirrhotic patients demonstrated substantially higher HCC incidence compared to non-cirrhotic individuals across both treatment groups. In the pre-matched cirrhotic cohort (n=471), interferon-based combination therapy provided significant protection against HCC development (log-rank p=0.002; **Supplementary Figure 4**). Kaplan-Meier analysis revealed early and sustained separation of cumulative incidence curves, with the treatment advantage emerging within the first 48 weeks and maintaining throughout the 6-year follow-up period.

After PSM of cirrhotic patients (n=408, 204 per group), the protective effect of IFN-based therapy remained statistically significant and clinically substantial (log-rank p=0.020; **Figure 4**). The absolute HCC incidence rates in the matched cirrhotic cohort were 18.6% in the NAs group versus 9.8% in the IFN group, representing an 8.8% absolute risk reduction - approximately 10-fold greater than observed in non-cirrhotic patients. The number needed to treat to prevent one HCC event in cirrhotic patients was 11 over 6 years, indicating markedly greater treatment efficiency in this high-risk population.

**Figure 4:**
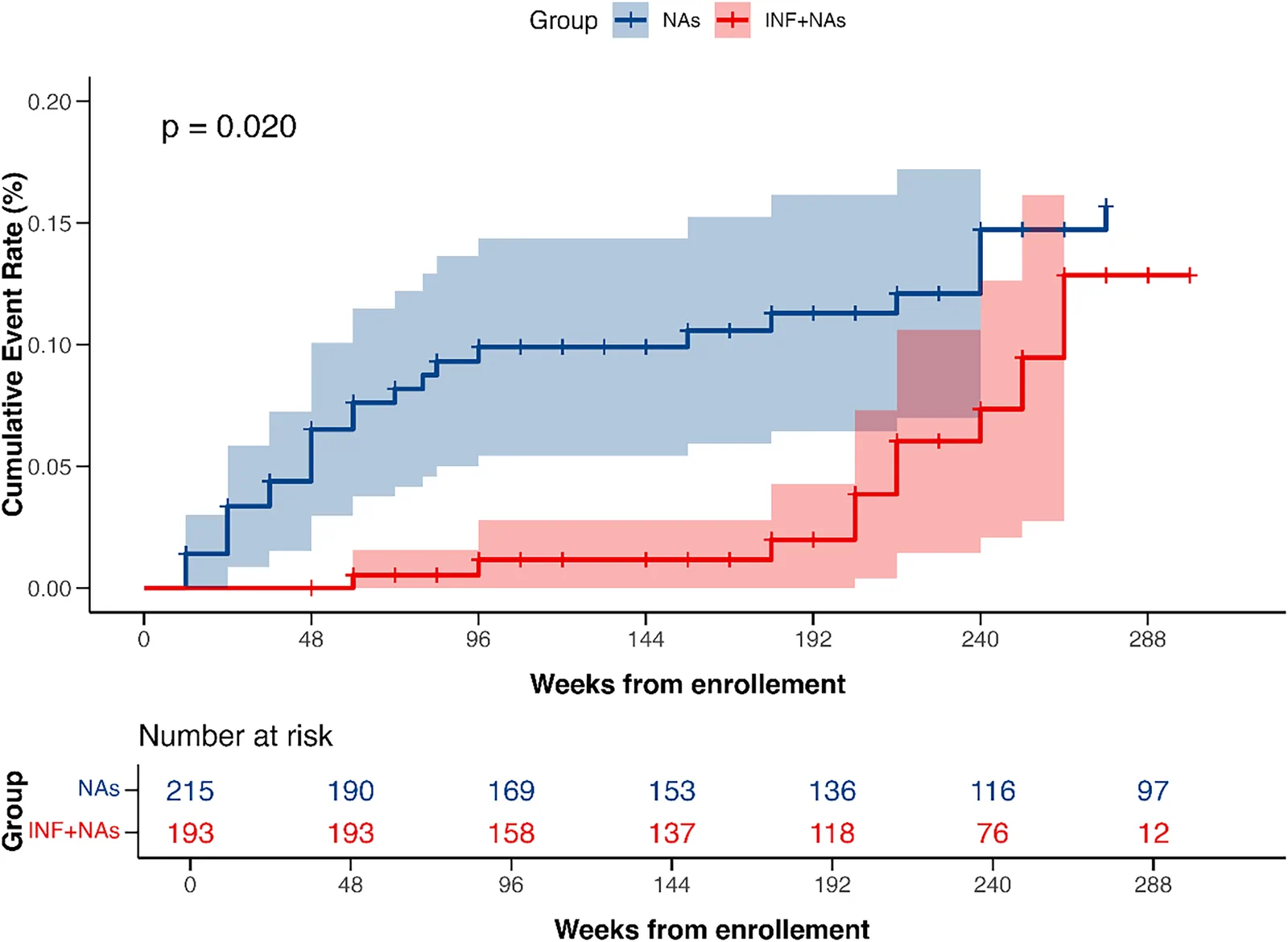
Kaplan-Meier analysis of HCC-free survival in cirrhotic patients after PSM. Cumulative incidence of hepatocellular carcinoma in cirrhotic patients receiving nucleos(t)ide analog (NAs) monotherapy (red line) versus interferon-based combination therapy (NAs + IFN, blue line) after propensity score matching (n=408, 1:1 matched). Log-rank test demonstrated significantly superior HCC-free survival in the NAs + IFN group (p = 0.020). Numbers at risk at each time point are shown below the figure.

The consistent treatment benefit observed in both pre- and post-matched analyses, despite reduced sample size, underscores the particular efficacy of IFN-based therapy in cirrhotic patients who face substantially elevated baseline HCC risk compared to their non-cirrhotic counterparts (15.2% vs 3.7% overall incidence).

#### Performance of established HCC risk prediction models in identifying HCC risk low HBsAg CHB patients with interferon-based therapy

##### Identifying truly low-risk patients

In the pre-matched cohort, all six established HCC prediction models (AASL-HCC, aMAP, CAMD, mPAGE-B, mREACH-B, and REAL-B) consistently demonstrated that patients classified as low-risk by these algorithms showed no significant differential treatment effect between IFN-based therapy and NAs monotherapy. As shown in **Supplementary Figure 5**, Kaplan-Meier analyses revealed no statistically significant differences in HCC incidence between treatment arms across all models: AASL-HCC low-risk: log-rank p = 0.169; aMAP low-risk: log-rank p = 0.546; CAMD low-risk: log-rank p = 0.810; mPAGE-B low-risk: log-rank p = 0.895; mREACH-B low-risk: log-rank p = 0.485; REAL-B low-risk: log-rank p = 0.657; The consistent non-significant findings across all six models in the pre-matched cohort suggest that currently available prediction tools effectively identify a patient subgroup with genuinely low HCC risk where treatment intensification with IFN provides no additional protective benefit. The convergence of these results across diverse algorithmic approaches indicates robust validation of these models’ ability to discriminate patients who may not require aggressive intervention beyond standard NAs therapy.

Consistent with pre-matched analyses, five of six established HCC prediction models (AASL-HCC, aMAP, CAMD, mPAGE-B, and REAL-B) demonstrated no significant treatment benefit from interferon-based therapy in their respective low-risk categories after propensity score matching. As shown in **Figure 5**, Kaplan-Meier analyses revealed non-significant differences in HCC incidence between treatment arms: AASL-HCC (log-rank p = 0.231), aMAP (p = 0.536), CAMD (p = 0.527), mPAGE-B (p = 0.692), and REAL-B (p = 0.632). This remarkable consistency across five independent validation models provides robust evidence that currently available risk stratification tools effectively identify patient subgroups with genuinely low HCC risk where treatment intensification offers no additional protective benefit.

**Figure 5:**
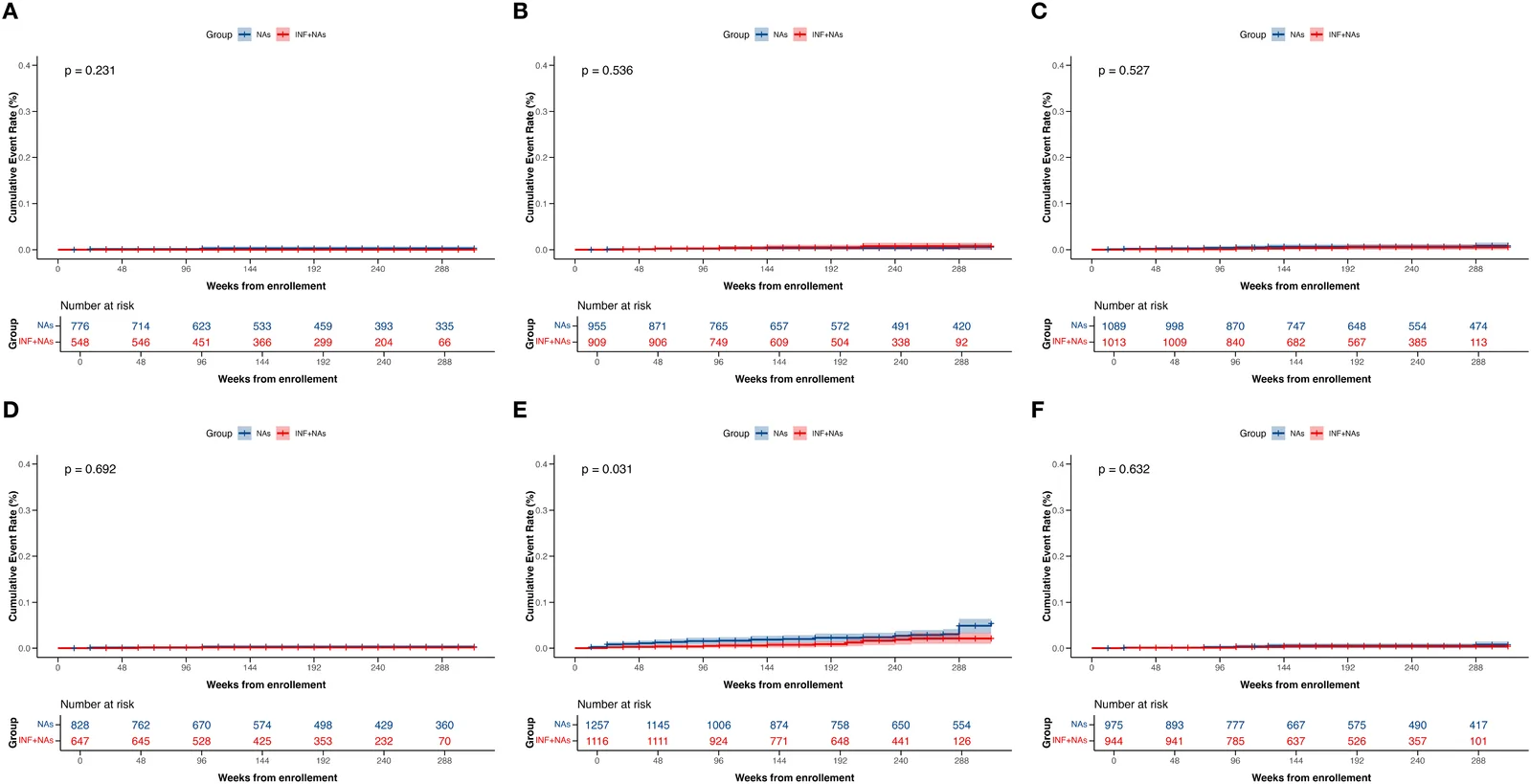
HCC risk stratification performance of six prediction models in propensity score-matched cohort. Kaplan-Meier analyses of hepatocellular carcinoma-free survival in patients classified as low-risk by six established prediction models after propensity score matching: **(A)** AASL-HCC, **(B)** aMAP, **(C)** CAMD, **(D)** mPAGE-B, **(E)** mREACH-B, and **(F)** REAL-B. Five models **(A-D, F)** showed no significant difference in HCC incidence between treatment arms (all p > 0.05), while mREACH-B **(E)** demonstrated significant benefit for IFN-based therapy even in its low-risk category (p = 0.031).

However, the mREACH-B model showed a divergent pattern in the matched cohort, with IFN-based therapy demonstrating significant protection even in its low-risk category (log-rank p = 0.031). This outlier finding suggests potential fundamental differences in mREACH-B’s risk classification methodology or cohort-specific characteristics that warrant further investigation. The discordance between mREACH-B and the other five models highlights the importance of multi-model validation in clinical risk stratification.

##### Identifying truly high-risk patients

All six established HCC prediction models (AASL-HCC, aMAP, CAMD, mPAGE-B, mREACH-B, and REAL-B) consistently demonstrated significant treatment benefits for interferon-based therapy in their respective intermediate-high risk categories within the pre-matched cohort. As shown in **Supplementary Figure 6**, Kaplan-Meier analyses revealed highly significant differences in HCC incidence favoring the IFN-based combination therapy across all models (all p < 0.001).

The remarkable consistency across all six prediction models provides robust evidence that currently available risk stratification tools effectively identify patient subgroups with genuinely elevated HCC risk who derive substantial benefit from treatment intensification with interferon-based therapy. The early separation of survival curves observed across all models suggests that the protective effect of IFN emerges within the first year of treatment and is maintained throughout the 6-year follow-up period.

The concordance of these findings across diverse algorithmic approaches - incorporating varying combinations of clinical, biochemical, and virological parameters - indicates that the identification of treatment-responsive high-risk patients is robust and not model-dependent. This consistency reinforces the clinical utility of these prediction tools for guiding treatment intensification decisions in chronic hepatitis B patients with intermediate to high HCC risk.

The significant treatment benefits of interferon-based therapy in intermediate-high risk patients, initially observed in the pre-matched cohort, were robustly maintained after propensity score matching across all six prediction models. As demonstrated in **Figure 6**, Kaplan-Meier analyses confirmed substantial HCC risk reduction with IFN-based combination therapy in properly matched intermediate-high risk patients identified by each model.

**Figure 6:**
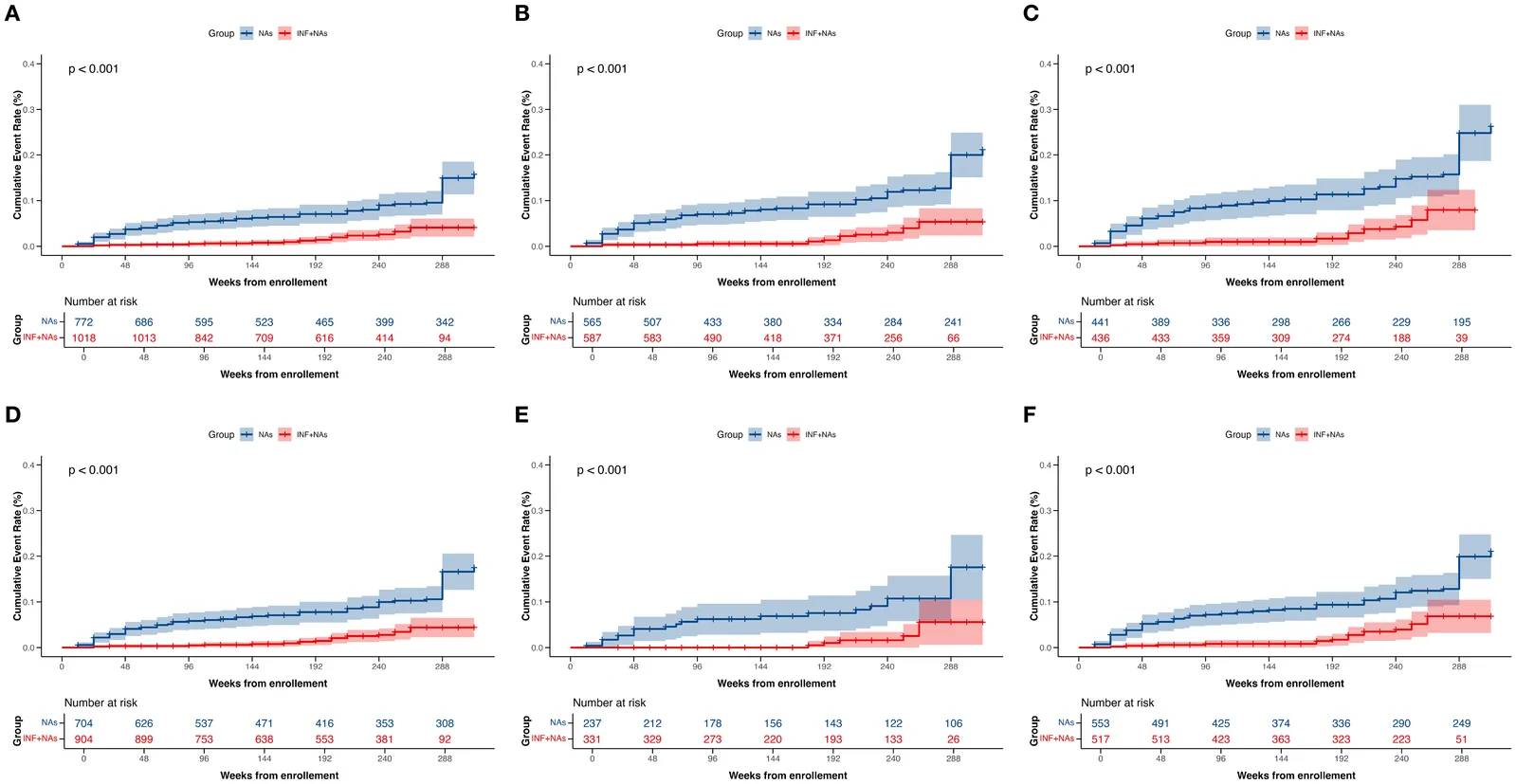
HCC risk stratification performance of six prediction models for intermediate-high risk patients in propensity score-matched cohort. Kaplan-Meier analyses of hepatocellular carcinoma-free survival in patients classified as intermediate-high risk by six established prediction models after propensity score matching: **(A)** AASL-HCC, **(B)** aMAP, **(C)** CAMD, **(D)** mPAGE-B, **(E)** mREACH-B, and **(F)** REAL-B. Five models **(A-D, F)** maintained exceptionally strong statistical significance (all p < 0.001), while mREACH-B **(E)** demonstrated slightly attenuated but still highly significant protection (p = 0.002). All models consistently identified patients who derived substantial benefit from IFN-based combination therapy.

Five of the six models (AASL-HCC, aMAP, CAMD, mPAGE-B, and REAL-B) maintained exceptionally strong statistical significance (all p < 0.001) in the matched cohort, with hazard ratios consistently favoring IFN-based therapy. The mREACH-B model, while showing a slightly attenuated effect size compared to the pre-matched analysis, still demonstrated highly significant protection (p = 0.002). This remarkable consistency across diverse prediction algorithms - incorporating varying combinations of clinical, biochemical, and virological parameters - provides compelling evidence that currently available risk stratification tools effectively identify patient subgroups who derive substantial benefit from treatment intensification.

The early and sustained separation of survival curves observed across all models suggests that the protective effect of IFN emerges within the first year of treatment and is maintained throughout the 6-year follow-up period. The convergence of findings across both pre- and post-matched analyses indicates that the treatment benefits are robust and not attributable to baseline confounding factors.

#### Differential HCC risk profiles by HBsAg serological response

##### HCC incidence by functional cure status

The study population was stratified by HBsAg serological response after antiviral therapy before and after PSM (**Tables 2**, **3**). Patients achieving functional cure (HBsAg seroclearance, n=526) and those with persistent antigenemia (n=2,364) showed comparable baseline characteristics before and after PSM, with minimal differences in demographic, clinical, and laboratory parameters.

**Table 2:** The descriptive results of patients with HBsAg turning negative.

Variable	Before PSM	After PSM
n	8	6
Age, year	64 (57, 80.75)	64 (55, 80.25)
Sex, female	2 (25.0%)	1 (16.7%)
BMI group		
0	0 (0.0%)	0 (0.0%)
1	5 (62.5%)	4 (66.7%)
2	3 (37.5%)	2 (33.3%)
3	0 (0.0%)	0 (0.0%)
Diabetes mellitus	2 (25.0%)	1 (16.7%)
Fatty liver	0 (0.0%)	0 (0.0%)
Family history of HepaB	5 (62.5%)	4 (66.7%)
Family history of HCC	2 (25.0%)	1 (16.7%)
Drink	2 (25.0%)	1 (16.7%)
Cirrhosis	5 (62.5%)	3 (50.0%)
AFP	3.10 (2, 15.59)	4.30 (2.75, 21.82)
ALT	26.50 (15, 35)	26.50 (17, 36.25)
WBC	5.54 (4.92, 6.42)	5.80 (4.98, 6.90)
PLT	151 (108.75, 228.75)	133 (103.75, 178)
NEUT	2.92 (2.52, 3.43)	3.01 (2.37, 3.92)
HGB	157 (148.50, 165)	157 (149.50, 165.50)
AST	30 (22.75, 38.25)	29.50 (20.50, 36.75)
ALB	46.24 (43.18, 48.20)	44.89 (42.90, 47.75)
GLB	30.55 (26.73, 33.93)	30.55 (26.87, 34.83)
GGT	54.50 (32.50, 94.25)	60.50 (31.75, 136.75)
TBIL	17.25 (11.63, 23.87)	16.75 (10.08, 24.35)
DBIL	4.10 (2.95, 4.98)	3.85 (2.63, 4.93)
HbsAg (QN)	62.99 (13.84, 256.73)	78.44 (11.13, 320.40)
GPR	0.50 (0.27, 1.30)	0.64 (0.30, 1.73)
FIB4 group		
1	2 (25.0%)	1 (16.7%)
2	4 (50.0%)	4 (66.7%)
3	2 (25.0%)	1 (16.7%)
APRI group		
1	4 (50.0%)	3 (50.0%)
2	4 (50.0%)	3 (50.0%)
3	0 (0.0%)	0 (0.0%)
Virus load > 2 log	4 (50.0%)	3 (50.0%)
Left liver thickness	107.50 (104.25, 114.75)	105 (102.25, 112.75)
Left liver anteroposterior diameter	60 (52, 64.50)	61.50 (48.50, 67)
Left liver lobe superior inferior dimension	61.50 (53.50, 73.25)	59.50 (51.75, 69.75)
Portal vein diameter	11 (10.85, 11.75)	11 (10.75, 11.25)
Spleen length	112.50 (109.75, 122.50)	112.50 (109, 123)
Splenic hilum vein diameter	5.50 (5, 7)	5 (5, 7.75)
HBsAg turning negative	8 (100.0%)	6 (100.0%)
HBsAb, positive	6 (75.0%)	4 (66.7%)
HBeAb, positive	8 (100.0%)	6 (100.0%)
ALT returning normal	5 (62.5%)	3 (50.0%)
ALT level rising	3 (37.5%)	3 (50.0%)
AFP at normal level	6 (75.0%)	4 (66.7%)

**Table 3:** The descriptive results of patients with HBsAg remaining positive.

Variable	Before PSM	After PSM
n	90	73
Age, year	65 (53, 73)	62 (51.50, 73)
Sex, female	10 (11.1%)	1 (1.4%)
BMI group		
0	2 (2.2%)	2 (2.7%)
1	64 (71.1%)	55 (75.3%)
2	24 (26.7%)	16 (21.9%)
3	0 (0.0%)	0 (0.0%)
Diabetes mellitus	13 (14.4%)	9 (12.3%)
Fatty liver	4 (4.4%)	4 (5.5%)
Family history of HepaB	44 (48.9%)	34 (46.6%)
Family history of HCC	13 (14.4%)	6 (8.2%)
Drink	12 (13.3%)	8 (11.0%)
Cirrhosis	56 (62.2%)	43 (58.9%)
AFP	3.45 (2, 16.82)	3 (2, 15.75)
ALT	29.50 (20, 38)	30 (21.50, 38.50)
WBC	5.92 (4.74, 6.76)	6.01 (4.80, 6.76)
PLT	170 (124.75, 213)	171 (138.50, 213)
NEUT	3.19 (2.23, 4.02)	3.26 (2.24, 4.14)
HGB	149.50 (136.50, 162.25)	152 (138.50, 163)
AST	29 (23, 39)	28 (23, 36)
ALB	44.90 (43, 47.58)	45.60 (43.45, 48.15)
GLB	29.30 (26.68, 31.72)	28.60 (26.45, 31.60)
GGT	34 (23, 51.25)	32 (23, 50)
TBIL	13.95 (11.48, 19.50)	13.60 (11.30, 17.13)
DBIL	3.75 (2.80, 5.25)	3.70 (2.80, 5.10)
HbsAg (QN)	601.20 (181.60, 1037.25)	502.60 (172.40, 999.50)
GPR	0.37 (0.20, 0.57)	0.32 (0.18, 0.54)
FIB4 group		
1	30 (33.3%)	27 (37.0%)
2	38 (42.2%)	32 (43.8%)
3	22 (24.4%)	14 (19.2%)
APRI group		
1	46 (51.1%)	39 (53.4%)
2	35 (38.9%)	27 (37.0%)
3	9 (10.0%)	7 (9.6%)
Virus load > 2 log	78 (86.7%)	61 (83.6%)
Left liver thickness	108 (101, 115.25)	108 (102.50, 115.50)
Left liver anteroposterior diameter	59.50 (52, 67)	58 (52.50, 66.50)
Left liver lobe superior inferior dimension	62 (49, 72.50)	67 (50, 75)
Portal vein diameter	11.70 (11, 12)	11.40 (11, 12)
Spleen length	109 (95.75, 123)	107 (93, 119.50)
Splenic hilum vein diameter	6 (5, 7)	6 (5, 7)
HBsAg turning negative	90 (100.0%)	73 (100.0%)
HBsAb, positive	86 (95.6%)	70 (95.9%)
HBeAb, positive	76 (84.4%)	61 (83.6%)
ALT returning normal	72 (80.0%)	59 (80.8%)
ALT level rising	18 (20.0%)	14 (19.2%)
AFP at normal level	62 (68.9%)	52 (71.2%)

As presented in **Table 4**, patients achieving functional cure demonstrated markedly reduced HCC risk compared to those with persistent antigenemia in both pre- and post-PSM analyses. In the matched cohort, HCC incidence was 1.14% (6/526) in the seroclearance group versus 3.09% (73/2,364) in the persistent HBsAg-positive group. The protective effect of seroclearance was particularly pronounced, with NAs-treated patients showing 12.44-fold higher HCC risk compared to IFN-treated patients in this subgroup (95% CI 2.24-69.19, p=0.004).

**Table 4:** The HCC percentage and ORs in different conditions before and after PSM.

	HCC cases Before PSM			HCC cases After PSM		
	n (%)	OR (95% CI)	P	n (%)	OR (95% CI)	P
All patient						
IFN	21 (1.2%)	1	–	20 (1.4%)	1	–
NAs	77 (4.9%)	4.27 (2.62 to 6.95)	<0.001	59 (4.1%)	3.03 (1.82 to 5.06)	<0.001
HBsAg remaining positive						
IFN	19 (1.6%)	1	–	18 (1.8%)	1	–
NAs	71 (4.7%)	3.08 (1.84 to 5.14)	<0.001	55 (4.0%)	2.27 (1.33 to 3.89)	0.003
HBsAg turning negative						
IFN	2 (0.3%)	1	–	2 (0.4%)	1	–
NAs	6 (7.3%)	22.82 (4.52 to 115.07)	<0.001	4 (5.3%)	12.44 (2.24 to 69.19)	0.004
HBsAg remaining positive and virus load < 2 log						
IFN	4 (2.5%)	1	–	4 (2.7%)	1	–
NAs	8 (2.7%)	1.09 (0.32 to 3.68)	0.890	8 (3.0%)	1.12 (0.33 to 3.80)	0.850
HBsAg remaining positive and virus load > 2 log						
IFN	15 (1.4%)	1	–	14 (1.7%)	1	–
NAs	63 (5.2%)	3.76 (2.13 to 6.64)	<0.001	47 (4.3%)	2.64 (1.45 to 4.83)	0.002

##### Predictors of HCC in functional cure patients

Multivariate analysis of the seroclearance subgroup (**Table 5**) identified advanced age (adjusted OR 1.31 per year, 95% CI 1.12-1.54, p<0.001), elevated AFP levels (aOR 1.17 per unit increase, 95% CI 1.04-1.31, p=0.008), and failure to achieve ALT normalization (aOR 0.01, 95% CI 0.00-0.49, p=0.019) as independent predictors of HCC. Although treatment modality showed strong univariate association (OR 0.08, p=0.004), it was not retained in the final multivariate model.

**Table 5:** The independent variables associated to HCC of patients with HBsAg turning negative after PSM.

	Univariate		Multivariate (forward: Wald test)	
Parameters	OR (95% CI)	P	OR (95% CI)	P
Group				
NAs	1	–		
IFN	0.08 (0.01 to 0.42)	0.004		
Age, year	1.19 (1.12 to 1.30)	<0.001	1.31 (1.12 to 1.54)	<0.001
DM	51.80 (2.20 to 638.83)	0.002		
Cirrhosis	9.00 (1.63 to 49.72)	0.008		
AFP	1.08 (1.01 to 1.14)	0.007	1.17 (1.04 to 1.31)	0.008
PLT	0.98 (0.97 to 0.99)	0.007		
GGT	1.02 (1.01 to 1.03)	0.001		
GPR	2.50 (1.13 to 4.71)	0.005		
FIB4 group				
1	1	–		
2	13.63 (1.99 to 268.04)	0.020		
3	17.50 (0.68 to 452.56)	0.045		
Spleen length	1.05 (1.00 to 1.08)	0.026		
HBsAb, positive	6.25 (1.21 to 45.50)	0.036		
ALT returning normal	0.11 (0.02 to 0.61)	0.008	0.01 (0.00 to 0.49)	0.019
AFP at normal level	0.10 (0.02 to 0.75)	0.010		

##### Risk factors of HCC in persistent HBsAg-positive patients

Among patients without seroclearance (**Table 6**), multivariate analysis revealed distinct risk factors including advanced age (aOR 1.15/year, 95% CI 1.12-1.18, p<0.001), diabetes mellitus (aOR 26.37, 95% CI 5.72-121.64, p<0.001), and cirrhosis (aOR 3.64, 95% CI 2.03-6.51, p<0.001). Protective factors included ALT normalization (aOR 0.30, 95% CI 0.14-0.63, p=0.001) and maintained normal AFP levels (aOR 0.18, 95% CI 0.08-0.37, p<0.001).

**Table 6:** The independent variables associated to HCC of patients with HBsAg remaining positive after PSM.

	Univariate		Multivariate (forward: Wald test)	
Parameters	OR (95% CI)	P	OR (95% CI)	P
Group				
NAs	1	–		
IFN	0.44 (0.25 to 0.74)	0.003		
Age, year	1.17 (1.14 to 1.20)	<0.001	1.15 (1.12 to 1.18)	<0.001
Sex	0.06 (0.00 to 0.25)	0.004		
DM	35.66 (13.50 to 94.28)	<0.001	26.37 (5.72 to 121.64)	<0.001
Cirrhosis	9.16 (5.68 to 14.94)	<0.001	3.64 (2.03 to 6.51)	<0.001
AFP	1.05 (1.03 to 1.06)	<0.001		
PLT	0.99 (0.99 to 1.00)	<0.001		
AST	1.01 (1.00 to 1.02)	0.019		
ALB	0.90 (0.84 to 0.96)	0.001		
GGT	1.01 (1.00 to 1.01)	<0.001		
TBIL	1.03 (1.00 to 1.05)	0.015		
FIB4 group				
1	1	–		
2	3.53 (2.10 to 5.99)	<0.001		
3	7.70 (3.83 to 14.88)	<0.001		
APRI group				
1	1	–		
2	3.13 (1.88 to 5.16)	<0.001		
3	7.02 (2.76 to 15.64)	<0.001		
Portal vein diameter	1.02 (0.99 to 1.05)	0.045		
ALT returning normal	0.43 (0.24 to 0.80)	0.005	0.30 (0.14 to 0.63)	0.001
AFP at normal level	0.10 (0.06 to 0.18)	<0.001	0.18 (0.08 to 0.37)	<0.001

##### Treatment efficacy across virological subgroups

As detailed in **Table 4** and graphically illustrated in **Figure 7**, IFN-based therapy demonstrated significant protection against HCC development across most patient subgroups. However, treatment response was modulated by viral replication status. In patients with persistent HBsAg positivity and low-level viremia (HBV DNA <2 log_10_ IU/mL), no significant treatment benefit was observed (post-PSM OR 1.12, 95% CI 0.33-3.80, p=0.850). Conversely, those with significant viremia (≥2 log_10_ IU/mL) derived substantial benefit from IFN-based therapy (post-PSM OR 2.64, 95% CI 1.45-4.83, p=0.002).

**Figure 7:**
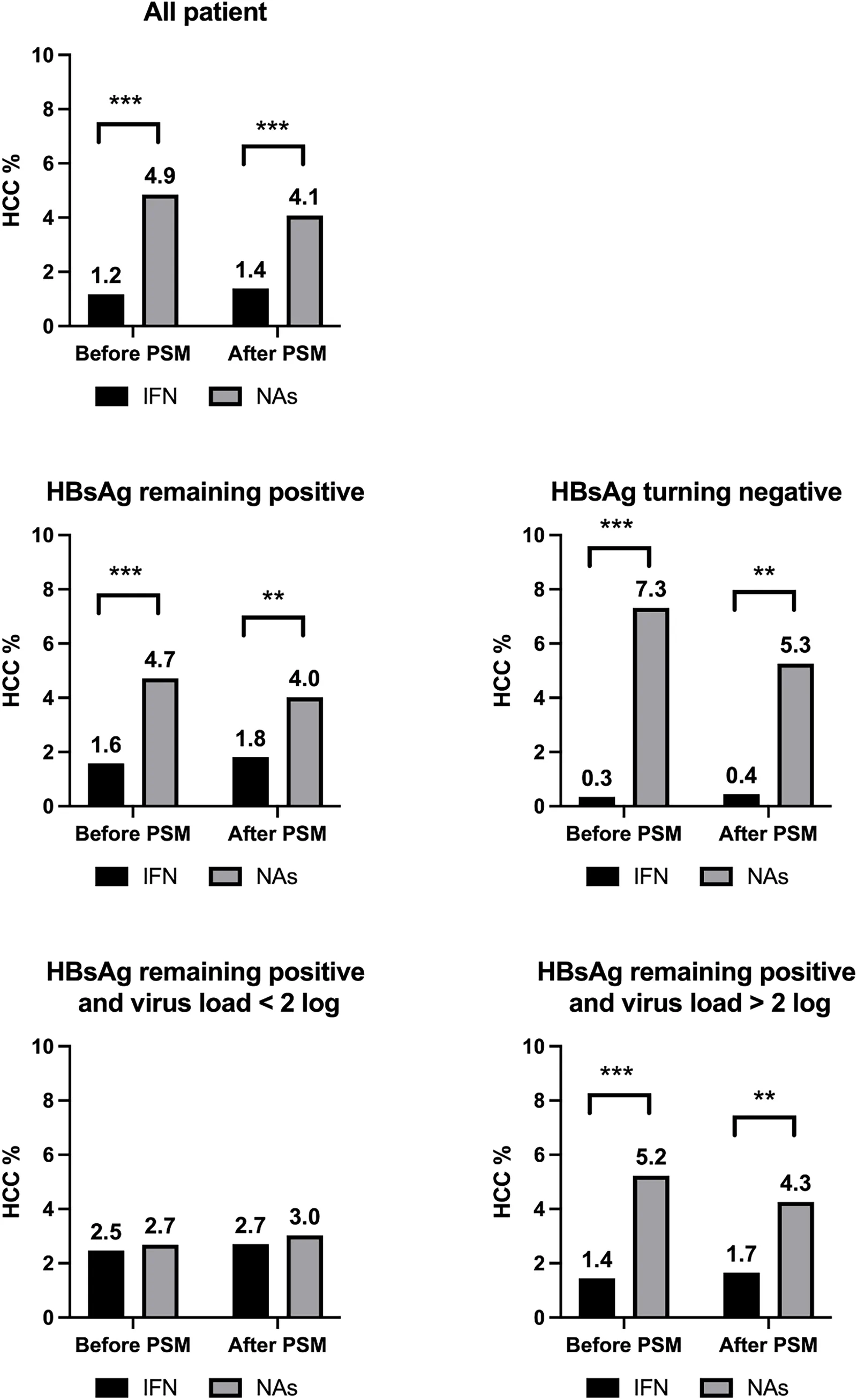
The HCC percentage (%) between IFN and NAs groups in different conditions before and after PSM. Significance levels are indicated by asterisks: *** denotes P<0.001, ** denotes P<0.01, * denotes P<0.05.

#### Development and validation of a dynamic HCC prediction model in IFN-treated patients

##### Model development and validation cohorts

The study cohort was randomly divided into derivation (n=1,010, 30.0%) and validation (n=2,353, 70.0%) sets at a 1:2 ratio. As shown in the baseline characteristics **Table 7**, both cohorts demonstrated excellent balance in demographic, clinical, and laboratory parameters, including treatment distribution (IFN-based therapy: 53.4% vs 52.6%), age distribution (median 42.0 years in both), cirrhosis prevalence (13.4% vs 14.3%), and key biochemical markers (all p>0.05), confirming comparable HCC risk profiles between groups.

**Table 7:** The baseline data of validation set and training set.

	Test N=1011	Training N=2356	P.overall
Group.1: Nucleoside 2 Interferon			0.705
Nucleoside	471(46.6%)	1116(47.4%)	
Interferon	540 (53.4%)	1240 (52.6%)	
Sex			0.135
female	188 (18.6%)	493(20.9%)	
Male	823(81.4%)	1863(79.1%)	
Age	42.0(36.0;49.0)	42.0(36.0;49.0)	0.161
Family history of HepaB			0.154
NO	541(53.5%)	1196 (50.8%)	
YES	470 (46.5%)	1160(49.2%)	
Family history of HCC			0.558
NO	937 (92.7%)	2168(92.0%)	
YES	74 (7.32%)	188 (7.98%)	
Alcohol History			0.264
NO	925 (91.5%)	2125(90.2%)	
YES	86(8.51%)	231 (9.80%)	
Liver Cirrhosis		6 (8.2%)	0.521
NO	876 (86.6%)	2020(85.7%)	
YES	135 (13.4%)	336 (14.3%)	
BMI-group			0.749
1	762 (75.4%)	1794 (76.1%)	
2	218(21.6%)	500 (21.2%)	
3	31 (3.07%)	62 (2.63%)	
ALT Group (1=Normal, 2=Elevated)			
1	756 (74.8%)	1794 (76.7%)	
2	255 (25.2%)	597 (25.3%)	
FIB4 group			0.694
1	725(71.7%)	1681 (71.3%)	
2	226 (22.4)%)	549 (23.3%)	
3	60 (5.93%)	126 (5.35%)	
APRI group			0.041
1	792 (78.3%)	1896 (80.5%)	
2	201 (19.9%)	397 (16.9%)	
3	18 (1.78%)	63(2.67%)	
AFP Normal (1=Yes)			0.616
0	52 (5.14%)	110 (4.67%)	
1	959 (94.9%)	2246 (95.3%)	
WBC	5.99 (4.95, 6.95)	5.97 (5.06, 7.05)	0.167
PLT	205(165, 243)	205 (169, 246)	0.273
NEUT	3.25 (2.54, 4.07)	3.26 (2.61, 4.05)	0.624
HGB	153 (142, 161)	153 (141, 161)	0.287
AST	25 (21, 30)	25 (21, 30)	0.576
ALB	47.4(45.5, 49.1)	47.2 (45.3, 49.2)	0.295
GLB	28.2(26, 30.7)	28.2(25.8, 30.5)	0.666
GGT	24 (17, 36)	24 (17, 36)	0.778
TBIL	12.2 (9.10, 16.5)	12.2 (9.20, 16.5)	0.816
DBIL	3.20(2.20, 4.60)	3.20 (2.30, 4.60)	0.606
HbsAg (QN)	405(130, 804)	384 (122, 754)	0.065
Right Liver Thickness	109(104, 115)	110(104, 116)	0.323
Left Liver A-P Diameter	55(46, 62)	55(46, 62)	0.864
Left Liver Craniocaudal:L	65(53, 76)	65(54, 76)	0.607
Portal Vein Diameter	11(11, 12)	11(10.7, 12.0)	0.103
Spleen Length	100(90, 111)	99(90, 110)	0.157
Splenic Vein Diameter	6(5, 6)	6(5, 6)	0.656

##### Predictor selection and nomogram construction

Using time-dependent Cox regression with feature selection (p<0.05), we identified eight independent predictors for HCC development: treatment modality (IFN vs NAs), AFP levels, family history of HCC, cirrhosis status, HBsAg quantitation, spleen length, age, and albumin levels. These variables were incorporated into a dynamic prediction nomogram (**Figure 8**) that calculates individual risk scores and estimates 3- and 5-year HCC probabilities.

**Figure 8:**
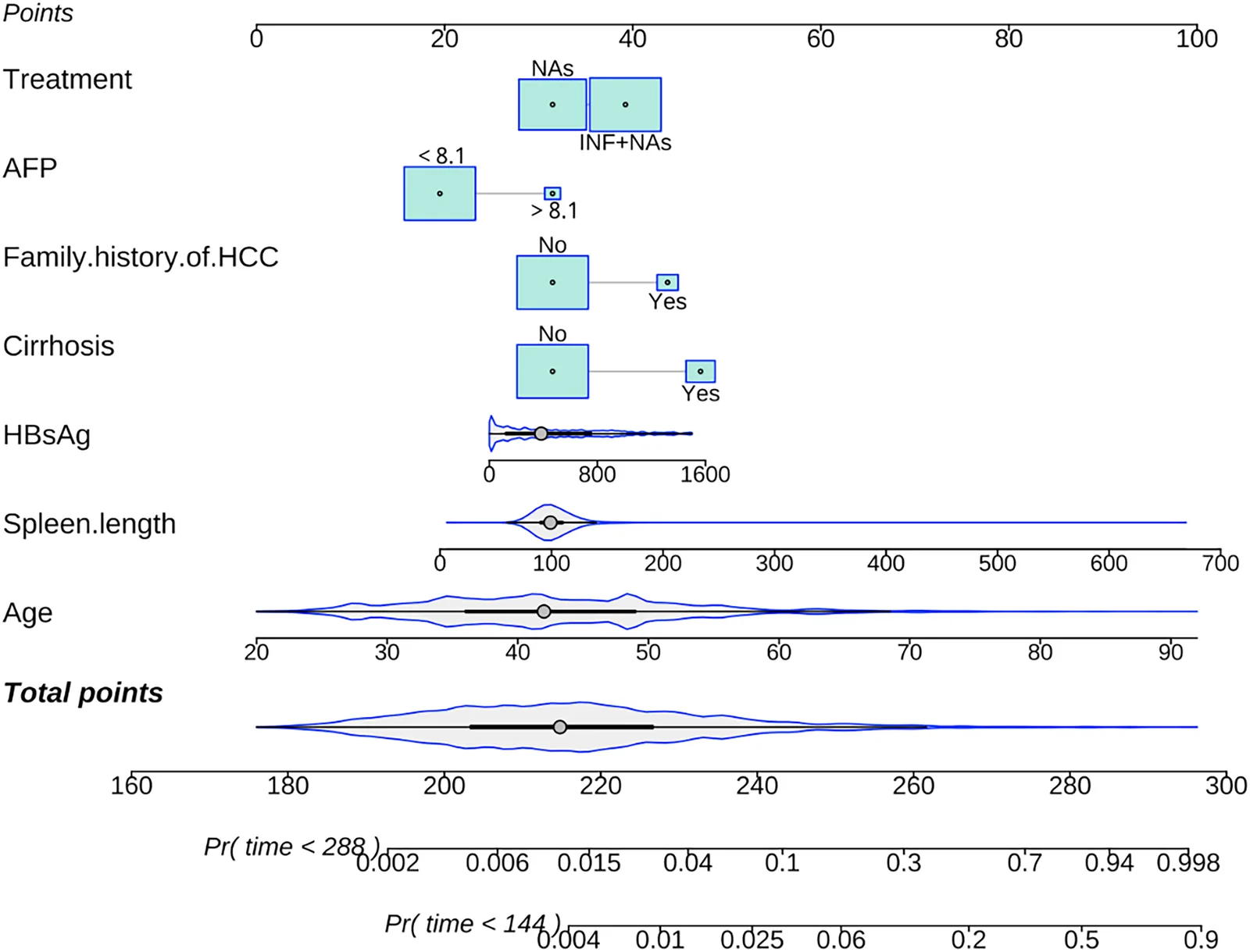
Nomogram for predicting hepatocellular carcinoma risk in patients achieving functional cure with interferon-based therapy. Dynamic prediction model incorporating eight clinically accessible parameters to estimate 3- and 5-year hepatocellular carcinoma risk in chronic hepatitis B patients achieving functional cure. The nomogram is used by summing points corresponding to each variable value (top scale), with total points projected to the bottom probability scales to obtain individualized risk estimates. The model demonstrates excellent discriminative ability with area under the curve values of 0.883 (95% CI: 0.813-0.952) for 3-year prediction and 0.888 (95% CI: 0.842-0.933) for 5-year prediction in the derivation cohort. HCC, hepatocellular carcinoma; IFN, interferon; NAs, nucleos(t)ide analogues; AFP, alpha-fetoprotein.

##### Model performance and validation

The nomogram demonstrated excellent discriminative ability in both derivation and validation cohorts. In the training set, the AUC reached 0.883 (95% CI: 0.813-0.952) for 3-year prediction and 0.888 (95% CI: 0.842-0.933) for 5-year prediction (**Supplementary Figure 7**). The validation set maintained strong performance with AUCs of 0.842 and 0.857 for 3- and 5-year predictions, respectively (**Supplementary Figure 8**), indicating robust generalizability of the prediction model.

#### Performance of established HCC prediction models in IFN-treated patients

##### Model performance in training cohort

All six established HCC prediction models demonstrated excellent discriminative ability in the derivation cohort of IFN-treated patients achieving functional cure (**Supplementary Figure 9**). The 3-year AUC values ranged from 0.820 to 0.888, while 5-year AUC values ranged from 0.826 to 0.889, indicating robust predictive performance across different time horizons. The aMAP and mPAGE-B models showed particularly strong performance, with 5-year AUCs exceeding 0.885.

##### Validation in independent cohort

Validation in the independent cohort confirmed maintained predictive accuracy for all models (**Supplementary Figure 10**). The 3-year AUC values ranged from 0.829 to 0.919, while 5-year AUC values ranged from 0.857 to 0.943. Notably, the mPAGE-B model achieved exceptional performance with AUCs of 0.919 (95% CI: 0.863-0.975) at 3 years and 0.943 (95% CI: 0.904-0.981) at 5 years, representing the highest predictive accuracy among all evaluated models.

#### Superior predictive accuracy of novel nomogram versus established prediction models across cohorts

Our newly developed nomogram demonstrated significantly superior predictive performance compared to all six established HCC prediction models in both derivation and validation cohorts across both time horizons (**Figure 9**). In the training cohort, it achieved AUCs of 0.883 (3-year) and 0.888 (5-year), representing significant improvements over existing models (p.adj <0.05 for most comparisons). In the validation cohort, the nomogram achieved outstanding discriminative ability with AUCs of 0.939 (95% CI: 0.890-0.987) for 3-year prediction and 0.956 (95% CI: 0.924-0.988) for 5-year prediction, significantly outperforming all comparator models (all p.adj <0.05 for 5-year prediction). Notably, the performance gap widened in the validation cohort, particularly for 5-year prediction, where our model showed 3.7-5.4% absolute improvement in AUC compared to the best-performing established models.

**Figure 9:**
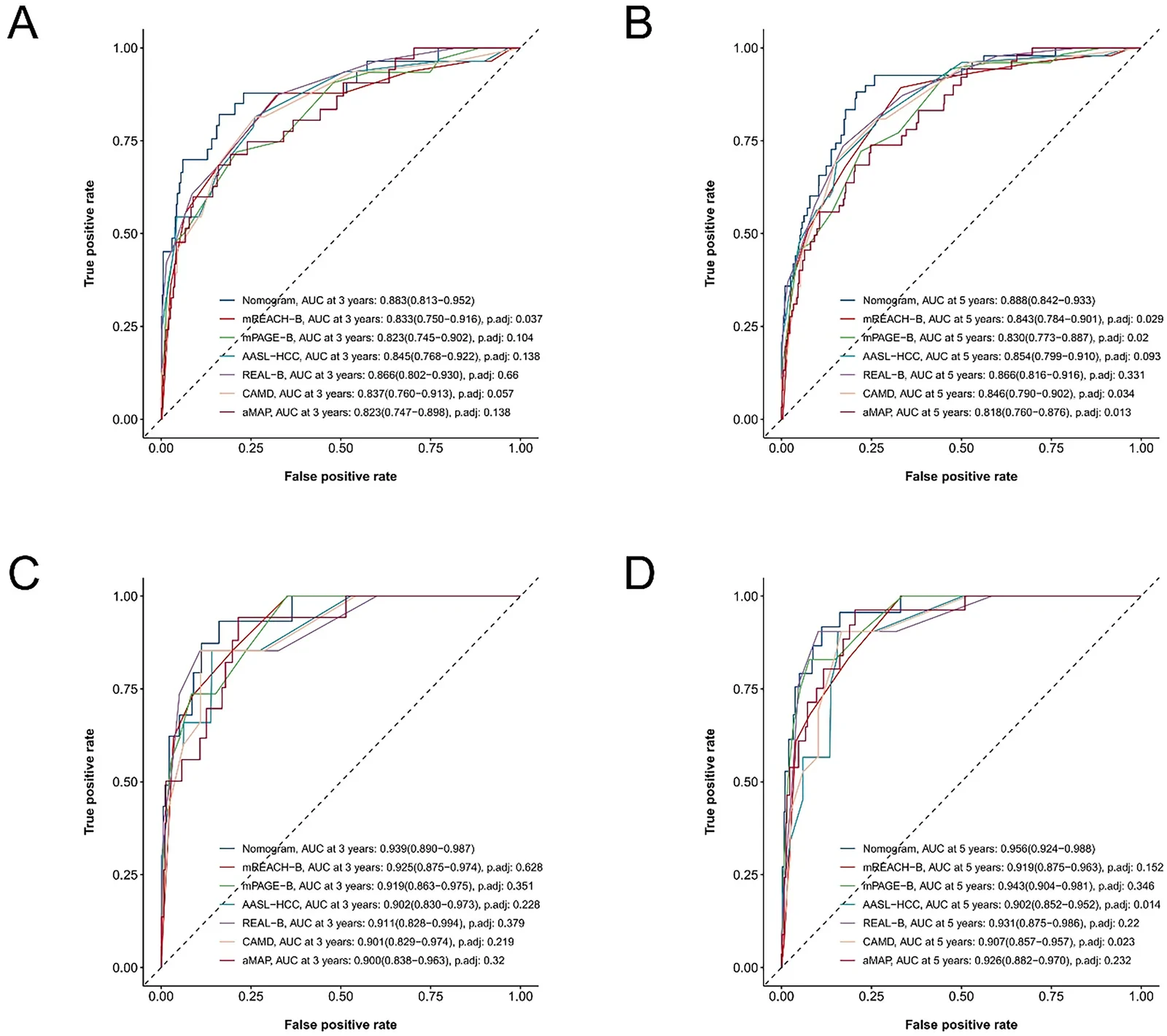
Comparative receiver operating characteristic analysis of hepatocellular carcinoma prediction models. **(A)** 3-year prediction performance in train cohort. **(B)** 5-year prediction performance in train cohort. **(C)** 3-year prediction performance in validation cohort. **(D)** 5-year prediction performance in validation cohort. *All AUC values presented with 95% confidence intervals. Statistical comparisons were adjusted for multiple testing using the Benjamini-Hochberg method.*.

## Discussion

This large-scale, retrospective cohort study demonstrates that interferon (IFN)-based combination therapy significantly reduces hepatocellular carcinoma (HCC) risk in chronic hepatitis B (CHB) patients with low baseline HBsAg levels, particularly among those with cirrhosis. The protective effect was robust even after rigorous propensity score matching, with a 3.03-fold lower HCC incidence in the IFN group compared to nucleos(t)ide analogue (NAs) monotherapy. Notably, the absolute risk reduction was most pronounced in cirrhotic patients (8.8%), corresponding to a number needed to treat (NNT) of only 11 over six years, underscoring the clinical efficacy of IFN-based regimens in this high-risk subgroup.

These findings align with and extend the growing body of evidence that functional cure-defined as sustained HBsAg loss-confers significant long-term clinical benefits, including reduced HCC risk (21, 22). However, our data further indicate that the pathway to functional cure matters: patients who achieved HBsAg clearance through IFN-based therapy had significantly lower HCC incidence than those who remained HBsAg-positive or even those with complete viral suppression on NAs alone (23, 24). This suggests that the immunomodulatory effects of IFN may confer additional anti-tumor benefits beyond virological control alone (25), possibly through restoration of immune surveillance (26), reduction of integrated HBV DNA (27, 28), or modulation of the tumor microenvironment (29, 30).

The differential effect of IFN was particularly evident in cirrhotic patients, who face a substantially elevated baseline HCC risk. Our data show that IFN-based therapy provided an 8.8% absolute risk reduction in this subgroup, far exceeding the benefit observed in non-cirrhotic patients. This finding is consistent with the concept that patients with advanced fibrosis or cirrhosis retain a residual oncogenic risk even after viral suppression, possibly due to persistent genetic alterations, epigenetic changes, or low-grade inflammation (31, 32). IFN’s pleiotropic effects-including antiproliferative, antiangiogenic, and immunostimulatory properties-may be particularly beneficial in this context (33).

We also evaluated the performance of six established HCC prediction models (AASL-HCC, aMAP, CAMD, mPAGE-B, mREACH-B, and REAL-B) in this unique population. Consistent with prior literature, most models effectively identified low-risk patients who derived minimal additional benefit from IFN intensification (34). However, all models consistently identified intermediate-to-high-risk patients who experienced significant HCC risk reduction with IFN-based therapy. The exception was mREACH-B, which showed a discordant pattern in the matched cohort, possibly due to its unique variable composition or cohort-specific characteristics (35). This underscores the importance of model-specific validation in distinct clinical settings and highlights the potential limitations of applying models derived from NA-treated or untreated cohorts to IFN-cured populations.

A key contribution of this study is the development and validation of a novel dynamic nomogram for HCC risk prediction specifically tailored to IFN-treated patients achieving functional cure. The model incorporates eight clinically accessible variables-treatment modality, AFP, family history of HCC, cirrhosis status, HBsAg quantitation, spleen length, age, and albumin-and demonstrated superior discriminative performance (AUC >0.88) compared to existing tools, both in derivation and validation cohorts. This tool may facilitate personalized post-treatment surveillance strategies and inform clinical decision-making regarding treatment intensification. For instance, a 55-year-old cirrhotic patient with elevated AFP and NAs monotherapy would have a 5-year HCC risk of ~40%, whereas a younger IFN-treated patient without risk factors would have a risk below 5%. Such granular risk stratification is crucial for optimizing resource allocation and individualizing patient follow-up (36).

Our findings are highly complementary to recent work by Yip et al. (23), who showed that HBsAg seroclearance on top of complete viral suppression further reduces HCC risk (aHR 0.24). However, unlike their study, which focused on NA-induced seroclearance, our cohort specifically examined IFN-based therapy, which may offer additional immunological benefits. Moreover, while Yip et al. found no reduction in hepatic events or liver-related mortality with HBsAg clearance, our data suggest that IFN-based therapy may benefit both HCC and possibly other liver-related outcomes, though this warrants further investigation.

Similarly, Yang et al. recently proposed a risk prediction model for HCC after HBsAg seroclearance based on age, cirrhosis, family history, and alcohol use (37). Our model expands upon this by incorporating treatment-specific variables (IFN vs. NAs), dynamic biomarkers (AFP, HBsAg), and imaging parameters (spleen length), making it particularly suitable for patients who achieved functional cure via IFN-based therapy. The superior performance of our nomogram (AUC 0.956 at 5 years) compared to existing models underscores the need for therapy-specific risk stratification tools.

Several limitations should be acknowledged. First, the retrospective design introduces potential unmeasured confounding despite propensity score matching. Second, the study was conducted at a single tertiary center in China, which may limit generalizability to other populations or healthcare settings. Third, the follow-up duration, though substantial, may not fully capture very late HCC events beyond six years. Fourth, we lacked data on HBV genotype, quantitative HBsAg levels, and detailed lifestyle factors, which may further refine risk prediction. Future prospective, multi-center studies with longer follow-up and integrated multi-omics approaches are warranted to validate these findings and explore underlying mechanisms.

In conclusion, IFN-based therapy significantly reduces HCC risk in CHB patients with low HBsAg, particularly in those with cirrhosis. Our proposed nomogram offers a validated, clinically useful tool for individualized risk stratification in this growing population of functionally cured patients, potentially guiding tailored surveillance and therapeutic strategies. As we move toward an era of finite-duration therapies aimed at functional cure, accurate risk prediction becomes increasingly important to ensure that the benefits of cure are maximized and the residual risks are managed effectively.

## Data Availability

The original contributions presented in the study are included in the article/**Supplementary Material**. Further inquiries can be directed to the corresponding authors.
