# Supplementary material for: Interferon-based HBV function cure therapy reduces hepatocellular carcinoma risk in low-HBsAg chronic hepatitis B patients: a propensity-matched cohort study and dynamic prediction model
Source: Front Immunol. 2026 Oct 19;17:1955742. doi: 10.3389/fimmu.2026.1955742 (PMC13724650; doi:10.3389/fimmu.2026.1955742)
Supplement: Supplementary file 1 [file Table1.docx]

**Supplementary table 1. The descriptive results between NAs and IFN treatment groups before PSM.**

| Variable | NAs | IFN | All | P |
| --- | --- | --- | --- | --- |
| n | 1587 | 1780 | 3367 |  |
| Age, year | 42 (36, 51) | 42 (36, 48) | 42 (36, 49) | <0.001 |
| Sex, female | 405 (25.5%) | 276 (15.5%) | 681 (20.2%) | <0.001 |
| BMI group |  |  |  | 0.289 |
| 0 | 2 (0.1%) | 0 (0.0%) | 2 (0.1%) |  |
| 1 | 1219 (76.8%) | 1337 (75.1%) | 2556 (75.9%) |  |
| 2 | 324 (20.4%) | 394 (22.1%) | 718 (21.3%) |  |
| 3 | 42 (2.6%) | 49 (2.8%) | 91 (2.7%) |  |
| Diabetes mellitus | 18 (1.1%) | 9 (0.5%) | 27 (0.8%) | 0.065 |
| Fatty liver | 30 (1.9%) | 518 (29.1%) | 548 (16.3%) | <0.001 |
| Family history of HepaB | 958 (60.4%) | 672 (37.8%) | 1630 (48.4%) | <0.001 |
| Family history of HCC | 180 (11.3%) | 82 (4.6%) | 262 (7.8%) | <0.001 |
| Drink | 291 (18.3%) | 26 (1.5%) | 317 (9.4%) | <0.001 |
| Cirrhosis | 275 (17.3%) | 196 (11.0%) | 471 (14.0%) | <0.001 |
| AFP | 2 (1, 3) | 2.28 (2, 3) | 2 (2, 3) | <0.001 |
| ALT | 26 (19, 36) | 25 (19, 34) | 25 (19, 35) | 0.091 |
| WBC | 5.95 (5.01, 6.94) | 6 (5.05, 7.09) | 5.98 (5.01, 7.03) | 0.297 |
| PLT | 195 (158, 243) | 212 (176, 247) | 205 (168, 245) | <0.001 |
| NEUT | 3.25 (2.59, 4.03) | 3.27 (2.58, 4.09) | 3.26 (2.59, 4.05) | 0.615 |
| HGB | 151 (137, 160) | 154 (145, 162) | 153 (141, 161) | <0.001 |
| AST | 26 (21, 32) | 24 (20, 28) | 25 (21, 30) | <0.001 |
| ALB | 46.20 (44.30, 48.20) | 48.10 (46.30, 49.80) | 47.30 (45.30, 49.20) | <0.001 |
| GLB | 28.20 (25.70, 30.60) | 28.20 (25.90, 30.60) | 28.20 (25.80, 30.60) | 0.670 |
| GGT | 25 (17, 38) | 23 (17, 34) | 24 (17, 36) | 0.028 |
| TBIL | 13.40 (10, 18.40) | 11.30 (8.60, 14.80) | 12.20 (9.20, 16.50) | <0.001 |
| DBIL | 3.80 (2.70, 5.40) | 2.70 (2, 3.80) | 3.20 (2.30, 4.60) | <0.001 |
| HbsAg (QN) | 395 (116, 801.30) | 384 (130, 754) | 391.20 (127.20, 782.90) | 0.822 |
| GPR | 0.21 (0.13, 0.37) | 0.20 (0.13, 0.29) | 0.20 (0.13, 0.32) | <0.001 |
| FIB4 group |  |  |  | <0.001 |
| 1 | 1065 (67.1%) | 1341 (75.3%) | 2406 (71.5%) |  |
| 2 | 372 (23.4%) | 403 (22.6%) | 775 (23.0%) |  |
| 3 | 150 (9.5%) | 36 (2.0%) | 186 (5.5%) |  |
| APRI group |  |  |  | <0.001 |
| 1 | 1179 (74.3%) | 1509 (84.8%) | 2688 (79.8%) |  |
| 2 | 342 (21.6%) | 256 (14.4%) | 598 (17.8%) |  |
| 3 | 66 (4.2%) | 15 (0.8%) | 81 (2.4%) |  |
| Virus load > 2 log | 1224 (77.1%) | 1406 (79.0%) | 2630 (78.1%) | 0.207 |
| Left liver thickness | 110 (104, 118) | 108 (104, 115) | 109 (104, 116) | <0.001 |
| Left liver anteroposterior diameter | 55 (46, 62) | 54 (46, 62) | 55 (46, 62) | 0.337 |
| Left liver lobe superior inferior dimension | 66 (53, 76) | 65 (54, 76) | 65 (54, 76) | 0.603 |
| Portal vein diameter | 11 (10.70, 12) | 11 (11, 12) | 11 (10.80, 12) | 0.002 |
| Spleen length | 100 (91, 111) | 98 (90, 109) | 100 (90, 110) | <0.001 |
| Splenic hilum vein diameter | 6 (5, 6) | 6 (5, 6) | 6 (5, 6) | 0.291 |
| HBsAg turning negative | 82 (5.2%) | 580 (32.6%) | 662 (19.7%) | <0.001 |
| HBsAb, positive | 1567 (98.7%) | 1206 (67.8%) | 2773 (82.4%) | <0.001 |
| HBeAb, positive | 1391 (87.6%) | 1522 (85.5%) | 2913 (86.5%) | 0.077 |
| ALT returning normal | 1471 (92.7%) | 1568 (88.1%) | 3039 (90.3%) | <0.001 |
| ALT level rising | 435 (27.4%) | 417 (23.4%) | 852 (25.3%) | 0.009 |
| AFP at normal level | 1511 (95.2%) | 1694 (95.2%) | 3205 (95.2%) | 1.000 |
| HCC | 77 (4.9%) | 21 (1.2%) | 98 (2.9%) | <0.001 |
